# Refinement of the classification of *DDX41* variants through analysis of aggregated clinical datasets

**DOI:** 10.1038/s41375-026-02886-6

**Published:** 2026-02-17

**Authors:** Ing Soo Tiong, Sally Hunter, Yamuna Kankanige, Nikita N. Mehta, Ryan A. Chisholm, Simon Wu, Jamilla Li, Joshua Casan, Kah Lok Chan, Lucy A. Godley, Lucy C. Fox, Piers Blombery

**Affiliations:** 1 Department of Pathology, Peter MacCallum Cancer Center, Melbourne, VIC Australia; 2 Sir Peter MacCallum Department of Oncology, University of Melbourne, Melbourne, VIC Australia; 3 Collaborative Centre for Genomic Cancer Medicine, Melbourne, VIC Australia; 4 Department of Pathology and Laboratory Medicine, Memorial Sloan Kettering Cancer Center, New York, NY USA; 5 Department of Biological Sciences, National University of Singapore, Singapore, Singapore; 6 Dorevitch Pathology, Melbourne, VIC Australia; 7 Department of Pathology, Queen Mary Hospital, Pok Fu Lam, Hong Kong; 8 Division of Hematology/Oncology, Department of Medicine, Robert H. Lurie Comprehensive Cancer Center, Northwestern University, Chicago, IL USA

**Keywords:** Genetic testing, Genetic testing

## Abstract

Deleterious germline *DDX41* variants are the leading cause of heritable predisposition to myelodysplastic neoplasia and acute myeloid leukemia (MDS/AML). Accurate classification of pathogenicity is crucial for managing patients and their families. The absence of specific guidelines, along with late-onset disease, incomplete penetrance, and founder variants, poses challenges in clinical and laboratory practice. We aggregated a synthetic cohort (ASC) of *DDX41* germline and somatic variants from 35 studies, including 1796 cases among 53686 patients, plus an additional 832 cases from non-cohort publications. We aimed to leverage the *DDX41*-ASC to develop and refine ACMG/AMP criteria on case enrichment *(PS4*), somatic associations (*PP4*), and computational prediction (*PP3/BP4*). Analysis confirmed that deleterious germline *DDX41* variants are most common in MDS/AML. A quasi-case-control study with ancestry matching revealed overestimated odds ratios for variants in underrepresented groups. Exploiting germline–somatic associations, we developed a Bayesian multinomial model that updates the odds of pathogenicity based on the presence and number of somatic patterns. Comparison of prediction tools showed that AlphaMissense outperformed REVEL in sensitivity. These results were integrated into an online tool to facilitate the consistent application of criteria. Overall, this comprehensive analysis of *DDX41*-ASC provides an evidence framework to inform the development of *DDX41*-specific curation guidelines.

## Introduction

Deleterious germline variants in *DDX41* are the most common cause of hereditary predisposition to myelodysplastic neoplasia (MDS) and acute myeloid leukemia (AML), accounting for ~80% of known germline predisposition cases and up to 5% of all newly diagnosed cases [1–8]. Testing for the presence of *DDX41* variants is now standard diagnostic practice [9–11] and included in many clinical sequencing panels used in the diagnosis of myeloid malignancies. Identification of causative germline *DDX41* variants has important clinical implications, including for diagnostic classification, disease prognosis, stem cell donor selection, prophylaxis for graft versus host disease, predictive testing of family members, and informing long-term monitoring strategies [8–18].

The diagnosis of *DDX41*-related hematologic malignancy predisposition syndrome (MONDO 0014809) relies on accurate classification of variant pathogenicity. *DDX41* variants are typically classified into five categories: benign (B), likely benign (LB), variant of uncertain significance (VUS), likely pathogenic (LP), and pathogenic (P) according to the joint American College of Medical Genetics and Genomics and Association of Molecular Pathology (ACMG/AMP) criteria [19]. This framework considers genomic, biological, functional, and population evidence. To further facilitate variant classification, the Clinical Genome Resource (ClinGen) provides gene-specific guidance on applying ACMG/AMP criteria. Clinical laboratories are encouraged to submit germline variant classifications to an open-source resource, ClinVar. To date, the Myeloid Malignancy Variant Expert Panel has specified curation rules for *RUNX1* [20, 21], but no such guidelines exist yet for *DDX41*, so individual laboratories lack consensus on variant classifications.

*DDX41* presents several challenges in variant interpretation as a result of: (i) late-onset disease, (ii) incomplete penetrance, (iii) the presence of founder variants, and (iv) the absence of validated functional assays. Conversely, *DDX41* also provides opportunities for unique contributions to pathogenicity, including the specificity of somatic findings as well as a large number of published cohort studies that focus on this group of patients. Given these challenges and opportunities in variant classification, we aimed to establish an aggregated synthetic cohort of *DDX41* variants (*DDX41*-ASC) from the published literature to study refinements to variant classification. Through this *DDX41*-ASC, we aimed to examine the connection between germline *DDX41* variants and disease contexts, conduct a comprehensive quasi-case-control analysis with ancestry group matching, apply novel statistical modeling to somatic variant data, and evaluate the performance of in silico tools for variant effect prediction. These results will lay the foundation for developing *DDX41* curation rules that can be applied internationally to ensure consistent variant classification worldwide.

## materials/subjects and methods

### Literature review

A literature review using the keyword “DDX41” on August 17, 2024, across PubMed, Medline, Web of Science, Scopus, and Embase identified 819 references, with an additional 17 through cross-referencing. Ultimately, 35 studies involving 53716 consecutive patients with hematological malignancies or cytopenias met the criteria for case-control series (Table S1A). Additionally, 595 cases from 55 more studies, and 250 cases from the Peter MacCallum Cancer Centre [17] were included for data on the somatic *DDX41* variant(s) (Table S1B). All reported *DDX41* variants were extracted using HGVSc nomenclature whenever possible and uniformly reclassified according to modified ACMG/AMP criteria, irrespective of the classifications reported in the source publications (Supplementary Methods).

### Quasi-case-control analysis

For cases reported in the literature, the reported ethnicity was used as a proxy for genetic ancestry (Table S1A). Population databases (controls) used for comparison against affected individuals with germline *DDX41* included the Genome Aggregation Database (gnomAD) v4.1.0 [22], ToMMo 54KJPN v20230626 (by the Tohoku Medical Megabank Organization) [23], and Korean Variant Archive v2 (KOVA) [24] (Supplementary Methods).

### Odds of pathogenicity

While we could apply the method of Maierhofer et al. [3] to infer the probability from (non-random) associations between germline and somatic variants using Bayesian reasoning, concern is warranted when applying this method to small samples of a specific germline variant under evaluation. Here, we developed a more stringent test for pathogenicity suitable for small sample sizes. For each germline variant, the posterior probability of observing different somatic *DDX41* variants was estimated via a multinomial distribution (Supplementary Methods). Odds of pathogenicity (OddsPath) are calculated from Tavtigian et al.’s formula, with evidence levels: very strong (≥350), strong (≥18.7), moderate (≥4.33), and supporting (≥2.08) [25]. All cases from published cohorts and our laboratory were included, regardless of diagnosis. To avoid double-counting somatic hit data from non-cohort studies, we included all cases without a somatic hit and only counted cases with unique somatic hits not reported in cohorts.

### Statistical analyses

The Fisher’s exact test was used to compare categorical variables, and the Wilcoxon or Kruskal-Wallis test was applied for numerical variables. Prevalence estimates were shown as point estimates with 95% confidence intervals (CIs). Odds ratios (OR) and 95% CIs were calculated with Haldane correction [26]. The lower bound of the 95% CI was used to determine the strength of pathogenicity based on log_10_(2.08) [25]. Decision trees for variant classification were built using recursive partitioning (rpart version 4.1.23; Supplementary Methods). Receiver operating characteristic (ROC) curves were employed to compare the performance of computational predictive tools (pROC version 1.18.5). Lollipop plots were created using ProteinPaint [27] with the protein domains based on Makishima et al. [2]. The analyses were performed using R version 4.4.1 (R Foundation for Statistical Computing, Vienna, Austria).

## Results

### Aggregation of existing DDX41 variant literature

**Fig. 1:**
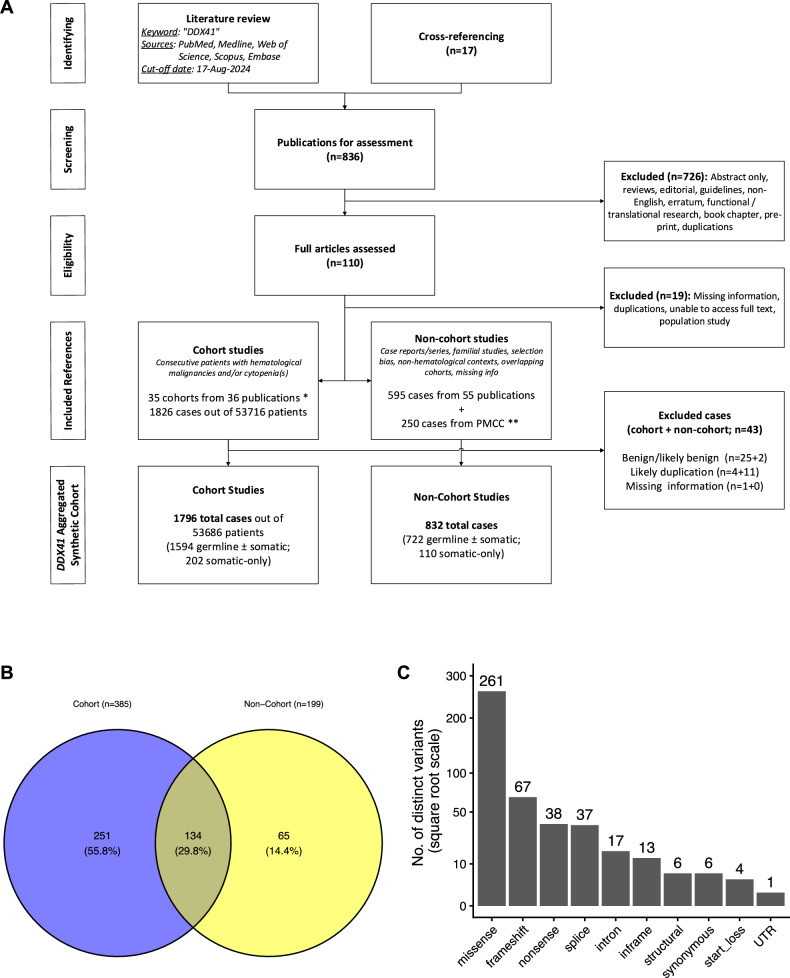
Summary of the *DDX41* aggregated synthetic cohort. **A** Flow diagram illustrating the literature review process for identifying published *DDX41* variants, with a cut-off date of 17-Aug-2024. *A single-center study cohort was reported in two separate publications. **Cases from the Peter MacCallum Cancer Centre (PMCC) were published in Wells et al. [17]. **B** Distribution of 450 distinct germline *DDX41* variants across the cohort sources. **C** Number of distinct *DDX41* variants by variant type.

After excluding B and LB variants, duplicate cases, and variants with incomplete information, the existing peer-reviewed literature (see Methods) was compiled into the *DDX41*-ASC, comprising 1796 cases with *DDX41* variants from 53686 consecutive patients, along with an additional 832 cases from non-cohort studies (Fig. 1A). 25% of cases were reported to have a germline origin confirmed. A total of 450 distinct variants were identified, including 65 variants (14%) found only in non-cohort studies (Fig. 1B). Missense variants showed the most diverse range with 261 different variants (including five with unclassified pathogenicity due to missing variant information), followed by frameshift (*n* = 67), nonsense (*n* = 38), and canonical splice site (*n* = 37) variants (Fig. 1C).

All variants are summarized and ranked using a recursive partitioning decision tree (Fig. S1). The co-occurrence of somatic *DDX41* hotspots (evidence code *PP4*) and enrichment in MDS/AML cases (*PS4)* were key criteria contributing to classification across all variant types. Five nonsense/frameshift variants were classified as LP based on the combination of *PVS1* and absence in population controls *(PM2*_supporting) [28]. Curation of missense variants using baseline approaches resulted in most variants (*n* = 233 [87%]) being classified as VUS.

Given the significant proportion of variants classified as VUS, we aimed to leverage the *DDX41*-ASC to develop and refine existing ACMG/AMP criteria concerning case enrichment (*PS4*), somatic associations (*PP4*), and computational prediction (*PP3/BP4*).

### Enrichment of DDX41 variants in MDS/AML (PS4)

To understand the prevalence of *DDX41* variants across different disease contexts, we included a subset of patients from the *DDX41*-ASC with a diagnosis of MDS/AML (*n* = 34051), other myeloid neoplasms (*n* = 8072), unexplained cytopenias (*n* = 5156), and lymphoid neoplasms (*n* = 1228); 5179 individuals with aplastic anemia or unspecified hematologic malignancies were excluded. Among the 1594 cases with a germline *DDX41* variant, we excluded 17 with aplastic anemia or healthy carriers, along with four cases with unclassified variants due to missing information, leaving 1573 cases included in the analysis.

**Fig. 2:**
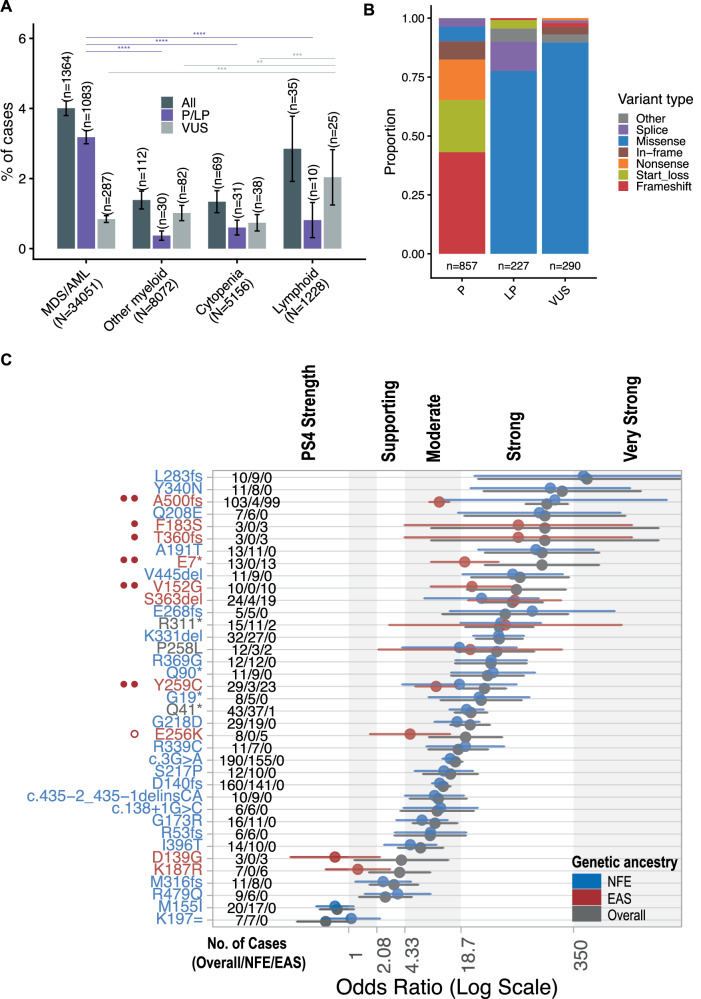
Association of germline *DDX41* variants with disease contexts and ancestral groups. **A** The proportion (%) of cases with pathogenic/likely pathogenic (P/LP) or uncertain (VUS) *DDX41* variants across various disease contexts in the literature cohorts. Whiskers indicate the 95% confidence interval. Twelve cases had two germline variants (6 with both VUS and P/LP were counted twice), and 7 cases had a lymphoid neoplasm in addition to either MDS/AML (*n* = 6) or another myeloid neoplasm (*n* = 1) (counted twice). Pairwise Fisher’s exact tests were adjusted using the Benjamini-Hochberg method. *P*-value annotations: <0.05 (*), < 0.01 (**), < 0.001 (***), < 0.0001 (****). **B** The stacked bar chart illustrates the distribution of 1374 germline *DDX41* variants in 1366 MDS/AML cases across different classes of pathogenicity. Two cases were unclassified due to missing HGVSc information and were excluded. Ten cases harbored two germline variants: 6 were VUS + P/LP, 3 were both VUS, and 1 was both LP. Variant counts for each classification are shown below each bar. **C** Quasi-case-control analysis of germline *DDX41* variants in patients with MDS/AML compared to population controls, differentiated by overall and ancestral groups (non-Finnish European [NFE] versus East Asian [EAS]). Variants with at least 10 total cases, 3 occurrences within the EAS ancestry group, and 5 NFE-only instances are included. The odds ratio and its 95% confidence interval are shown. Gray, blue, and red represent overall, NFE, and EAS ancestry groups, respectively, based on the exclusivity (or lack thereof) of variants within each ancestry group and the control group. Red circles to the left of the variant indicate downgraded variants due to added ancestry matching: *PS4*_moderate (two solid circles), *PS4*_supporting (single solid circle), or *PS4*_notmet (open red circle).

Overall, a germline P/LP/VUS variant was found in 4.0% of MDS/AML cases, 2.9% of lymphoid neoplasms, 1.4% of other myeloid neoplasms, and 1.3% of cases with cytopenias (Fig. 2A). Notably, 7 cases had a diagnosis of lymphoid neoplasm in addition to MDS/AML (*n* = 6) or another myeloid neoplasm (*n* = 1), and 6 MDS/AML cases had two germline variants, each being P/LP and VUS. A germline P/LP variant was found in 3.2% of MDS/AML cases (95% CI: 3.0–3.4%), which is significantly higher than in other diseases. The presence of a *DDX41* VUS was more common in lymphoid neoplasms (2%) compared to other diagnoses (0.7 to 1.0%). Among all reported germline variants, patients with MDS/AML had the highest proportion of P/LP variants (79%), followed by cases with unexplained cytopenias (45%), lymphoid neoplasms (29%), and other myeloid neoplasms (26%).

Among 1366 MDS/AML cases, we identified 1084 P/LP, 290 VUS, and 2 unclassified (missing HGVSc) germline *DDX41* variants; 10 cases carried two germline variants (6 were VUS + P/LP, 3 were both VUS, and 1 was both LP). In total, 1374 variants corresponding to 324 distinct changes are summarized in Fig. S2. The most common types of P variants were frameshift (43%), start-loss (22%), and nonsense (17%). Missense variants were the most prevalent variant type in LP and VUS, accounting for 77.5% and 90% of cases, respectively (Fig. 2B).

Cases involving other myeloid neoplasms (myeloproliferative neoplasm [MPN; *n* = 50], MDS/MPN [*n* = 13], unspecified [*n* = 48]) or unexplained cytopenias (*n* = 69) revealed 103 distinct germline variants, with the M155I variant being the most common (8%). Notably, 70 variants occurred only once (Fig. S3A), and 10 cases had somatic-only *DDX41* variants. There was a limited number of patients with lymphoid neoplasms across seven studies: 25 with acute lymphoblastic leukemia and 17 mature B-cell neoplasms [1, 7, 27, 29–32]. Of these, 35 had a germline variant, whereas seven had somatic-only *DDX41* variants (Fig. S3B). Eight also had myeloid neoplasms: MDS/AML (*n* = 7) and therapy-related MDS/MPN (*n* = 1). The R164W variant, previously speculated to be associated with lymphoma [33], was found in three patients: lymphoplasmacytic lymphoma (LPL) with pancytopenia, gamma heavy chain disease/*MYD88*-negative LPL, and chronic lymphocytic leukemia (*n* = 1 each).

#### Ancestry group-specific variability of DDX41 variant enrichment (PS4)

After confirming the significant association between germline *DDX41* variants and MDS/AML, we conducted a quasi-case-control study of specific variants in MDS/AML cases versus population controls (see “Methods”). We focused on variants with at least ten cases, three occurrences in the East Asian (EAS) ancestry group, and/or five non-Finnish European (NFE)-only instances (Fig. 2C and Table S2). The odds ratios for NFE-specific variants were generally consistent across overall and ancestry-specific data, except when NFE had a lower allele frequency than another group, such as for R479Q (0.03% in Admixed American versus 0.003% in NFE).

Using gnomAD total allele counts overestimated the OR of EAS ancestry variants. As a result of added ancestry matching, the *PS4* criterion strengths were adjusted: from strong to moderate (A500fs, E7*, V152G, Y259C), moderate to supporting (F183S, T360fs), and moderate to not met (E256K) (Fig. 2C).

Overall, eight variants showed a strong association with MDS/AML, with the lower bound of the 95% CI ≥ 18.7: L283fs, Y340N, Q208E, A191T, V445del, S363del, R311*, and K331del. The two most common germline variants, c.3G>A and D140fs, were moderately enriched in MDS/AML cases (≥4.33), along with 12 other variants. Three variants met the *PS4*_supporting criterion (≥2.08).

### Characteristics of somatic DDX41 variants (PP4)

The presence of somatic *DDX41* variants is a characteristic feature in *DDX41*-related hematologic malignancy predisposition syndrome [3]. We further characterized the observed somatic variants using the *DDX41*-ASC, which includes 34051 individuals with MDS/AML, of whom 1552 cases had a *DDX41* variant, including 828 with both germline and somatic variants, 538 with germline-only, and 186 with somatic-only *DDX41* variants.

**Fig. 3:**
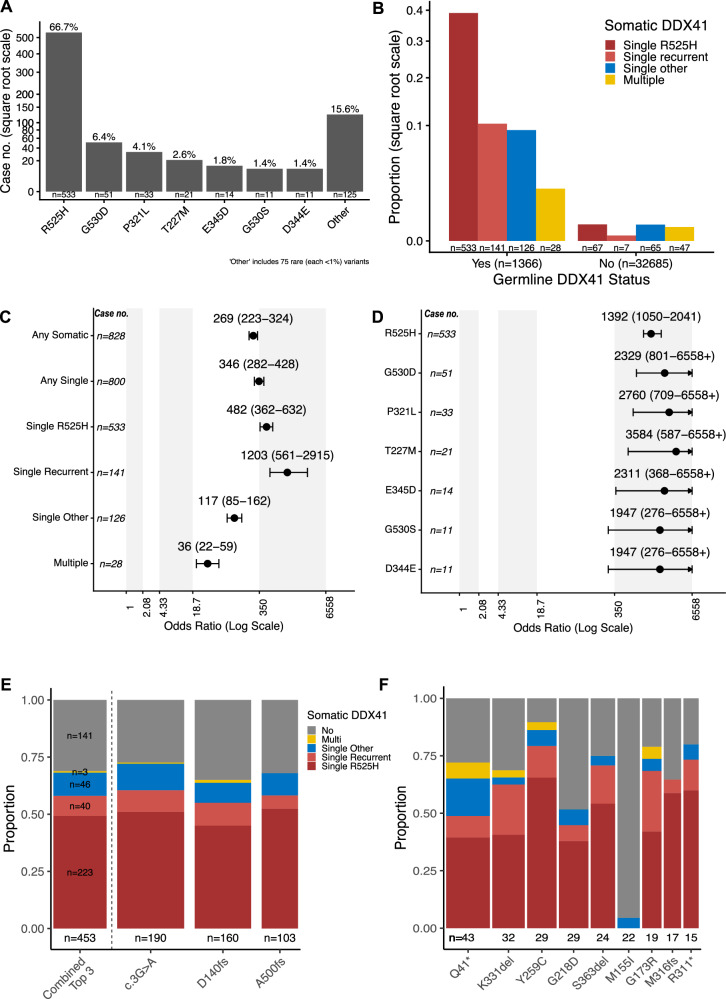
Characteristics of somatic *DDX41* variants in myelodysplastic neoplasia/acute myeloid leukemia (MDS/AML). **A** The frequency of single *DDX41* somatic variants observed alongside a germline *DDX41* variant in 799 cases of MDS/AML. One case was excluded due to missing variant information. **B** The proportions of somatic *DDX41* variant types among cases with or without a germline *DDX41* variant. **C**, **D** The association between various somatic *DDX41* variants and germline *DDX41* variants in MDS/AML. Odds ratios (ORs) and 95% confidence intervals were calculated from a quasi-case-control study. Higher ORs indicate a higher prevalence of somatic variants among patients with MDS/AML who carry a germline *DDX41* variant. Case counts represent the number of cases with each somatic variant type. **E**, **F** The correlation between the most prevalent germline *DDX41* variants (occurring in at least 15 instances) and the types of somatic *DDX41* variants.

We initially analyzed MDS/AML cases that included both germline and single somatic *DDX41* variants (*n* = 799 cases; one excluded for missing data). As expected, R525H was the most common somatic variant (*n* = 533; 66.7%). This was followed by six recurrent missense variants: G530D (c.1589G>A; 6.4%), P321L (c.962C>T; 4.1%), T227M (c.680C>T; 2.6%), E345D (c.1035G>C or c.1035G>T; 1.8%), G530S (c.1589G>A; 1.4%), and D344E (c.1032C>G or c.1032C>A; 1.4%) (Fig. 3A). The remaining 15.6% of cases harbored 75 different *DDX41* variants, each with up to six instances in less than 1% of cases. Missense variants were the most common, comprising 99% of all somatic variants; six were in-frame variants, and three were truncating variants (Fig. S4A). We observed significant differences in the median variant allele fractions (VAFs) among the somatic *DDX41* variants (Fig. S4B): P321L (23%), T227M (13.5%), D344E (9.7%), E345D (9.5%), G530D (8.9%), R525H (7.5%), and G530S (6%).

We then examined the frequency of different types of somatic *DDX41* variants among cases with and without a germline *DDX41* variant (P, LP, and VUS). Among 1366 individuals with MDS/AML and a germline *DDX41* variant, 538 (39%) had none, 800 (58.6%) had one, 27 (2%) had two, and one had three somatic *DDX41* variants. In contrast, among the 32685 cases of MDS/AML without an identified germline *DDX41* variant, somatic *DDX41* variants were rarely found: a single variant in 139 cases (0.43%) of which 74 (0.23%) were a recurrent hotspot, and multiple variants in 47 cases (0.14%) (Table S3). In cases without a germline *DDX41* variant where somatic *DDX41* variants were present, they tended to be single non-recurrent or multiple variants compared to those with a germline *DDX41* variant (Table S3 and Fig. 3B). Indeed, a single somatic *DDX41* variant was strongly linked to a germline P/LP/VUS *DDX41* variant (OR = 346, 95% CI: 282–428) (Fig. 3C). This association was even stronger when R525H was considered alone (OR = 482, 95% CI: 362–632) or only recurrent non-R525H (OR = 1203, 95% CI: 561–2915) somatic variants (Fig. 3D). Other single non-recurrent or multiple somatic variants remained significantly associated with a germline variant, though to a lesser degree (Fig. 3C).

The association between recurrent somatic *DDX41* variants was consistent across the well-established P/LP germline *DDX41* variants (Fig. 3E, F). Among the three most frequent germline *DDX41* variants—c.3G>A, D140fs, and A500fs (combined *n* = 453)—a single somatic R525H variant was found in 45–52% of cases, single recurrent non-R525H missense variant in 6–10%, single non-recurrent variant in 9–12%, and multiple somatic variants in 0–1% (Fig. 3E). This pattern is similarly observed for other less common but recurring germline P/LP *DDX41* variants (Fig. 3F). In contrast, the M155I variant (classified as a VUS; gnomAD frequency 0.04%) was observed only once with a single non-recurrent somatic *DDX41* variant. The association with other less common germline variants is summarized in Fig. S5.

The variant details of 186 cases of MDS/AML with somatic-only *DDX41* variants are summarized in Fig. S6. Among 139 cases with a somatic-only *DDX41* variant, 91% were missense, but recurrent non-R525H missense variants were rare. One case had three somatic-only *DDX41* variants: L87V, Y451C, and G586R. The remaining 46 cases had two somatic-only variants: one missense (R525H in 67%) or in-frame, combined with either another missense/in-frame or truncating variant, with both combinations occurring equally (Fig. S6). When seen as single or double variants, the VAFs of (assumed) somatic-only *DDX41* variants were similar to those with a germline variant (Fig. S7).

Overall, these findings support a specific association between deleterious *DDX41* variants and the pattern of somatic second hits. In contrast, although somatic-only *DDX41* variants can occur, they are much rarer and have different variant profiles.

#### Odds of pathogenicity from somatic DDX41 variants (PP4)

**Fig. 4:**
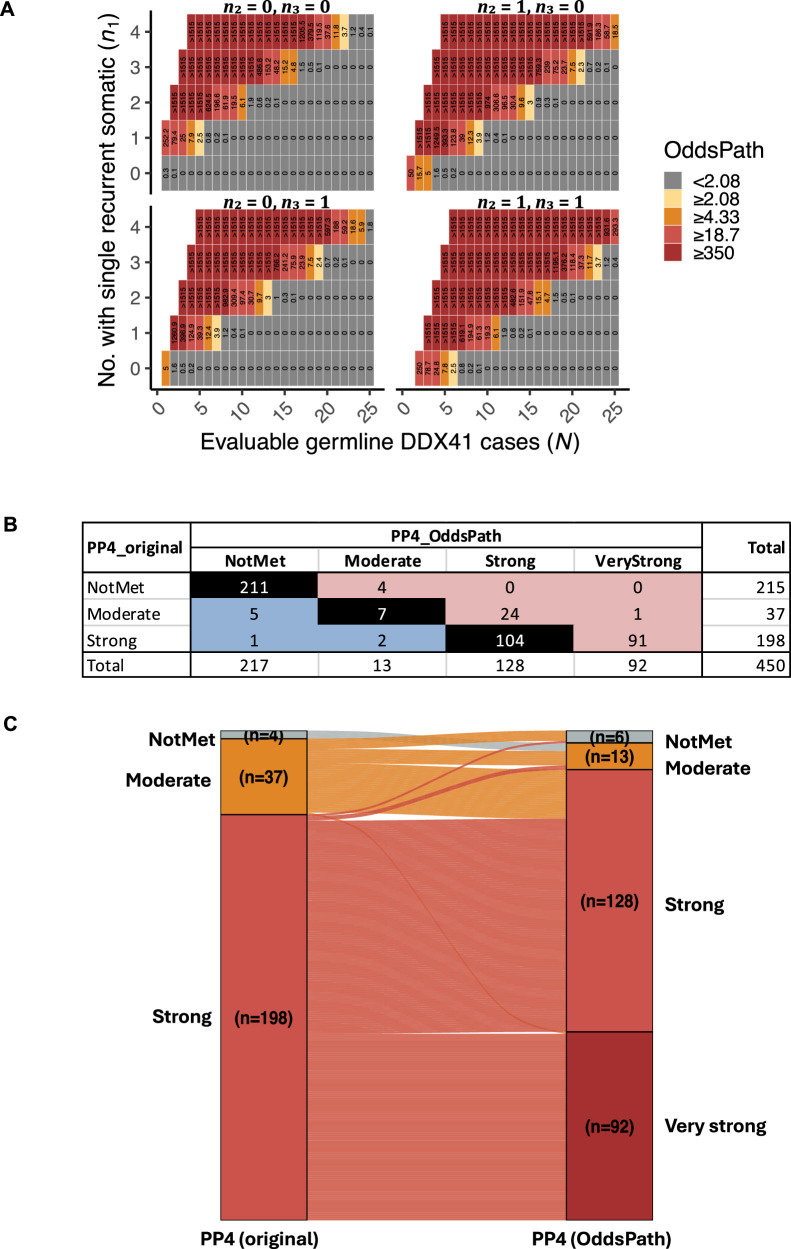
Odds of pathogenicity (OddsPath) based on the multinomial probability distribution of somatic *DDX41* variants. **A** Simulation of OddsPath based on the observed counts of single recurrent (R525H and non-R525H) somatic missense variants (*n*_1_), single non-recurrent somatic variants (*n*_2_), and multiple somatic hits (*n*_3_) among up to 25 evaluable cases (*N*) of germline *DDX41* variants. **B**, **C** Contingency table and Sankey diagram comparing evidence strength levels between the original approach (modified from Maierhofer et al. [3] and OddsPath. In the Sankey diagram, 211 cases that do not meet both criteria are excluded.

After observing a strong non-random association between germline and somatic *DDX41* variants and building on previous work [3], we sought to evaluate the pathogenicity of germline *DDX41* variants informed by the presence, number, and pattern of somatic variants. In cases of MDS/AML, the three most common pathogenic variants (c.3G>A, D140fs, and A500fs) were observed to have single recurrent missense (both R525H and non-R525H), single non-recurrent, and multiple somatic variants in 58%, 10%, and 0.7% of cases, respectively (Fig. 3E). In contrast, these somatic patterns were observed in 0.23%, 0.20%, and 0.14% of cases without a germline *DDX41* variant (Fig. 3B). We calculated the posterior probability of pathogenicity and OddsPath [25] using a multinomial probability mass function, noting that an observation of an isolated case with a recurrent somatic hit has a posterior probability of 97% and an OddsPath of 252 (Fig. 4A), equivalent to a “strong” level of evidence for pathogenicity in the modifications suggested to the ACMG/AMP evidence framework [25, 34].

Details of somatic occurrences of all 450 distinct germline *DDX41* variants are provided in Table S4. Overall, 92 variants had an OddsPath ≥350, consistent with a “very strong” level of evidence (Fig. 4B, C). Twenty-five variants were upgraded from *PP4*_moderate to *PP4*_strong (*n* = 24) or very strong (*n* = 1): five from recognizing additional somatic hotspots, and the remaining from non-recurrent single or multiple somatic hits.

In contrast, eight variants had evidence downgraded based on OddsPath (Table S4). These included six with OddsPath <2.08: M155I (*n* = 39), K187R (*n* = 19), R219H (*n* = 12), R339L (*n* = 6), R525H (*n* = 5), and P321L (*n* = 5). For R525H or P321L, only two of five cases each had confirmed germline origin. Two variants (I207T and c.138+5G>T) were downgraded from *PP4*_strong to *PP4*_moderate because only one somatic hotspot was observed out of four evaluable cases. Notably, M155I and R219H had three and two single non-recurrent somatic hits, respectively, a pattern highly unlikely due to chance despite a low OddsPath (Fig. S8).

When calculating the OddsPath, it is essential to consider all evidence of somatic occurrences. Incorporating non-cohort cases resulted in a total of 55 upgrades, including 40 variants from an OddsPath of <2.08, to *PP4*_moderate (*n* = 1), strong (*n* = 33), and very strong (*n* = 6).

#### In silico tool comparison for missense variants (PP3/BP4)

Given the numerous missense *DDX41* variants classified as VUS, we assessed the REVEL score [35] for missense variants and compared it with AlphaMissense [36]. After removing the *PP3* criterion, only 21 missense variants remained as P/LP. Therefore, we used all germline missense variants co-occurring with a single recurrent somatic hit to create a pathogenic truth set (*n* = 61), excluding those within the splice junctions. We retrieved 678 missense variants from gnomAD v4.1.0. After curation, only four were LB, so we included 503 in the benign truth set, excluding 171 found in the *DDX41*-ASC.

**Fig. 5:**
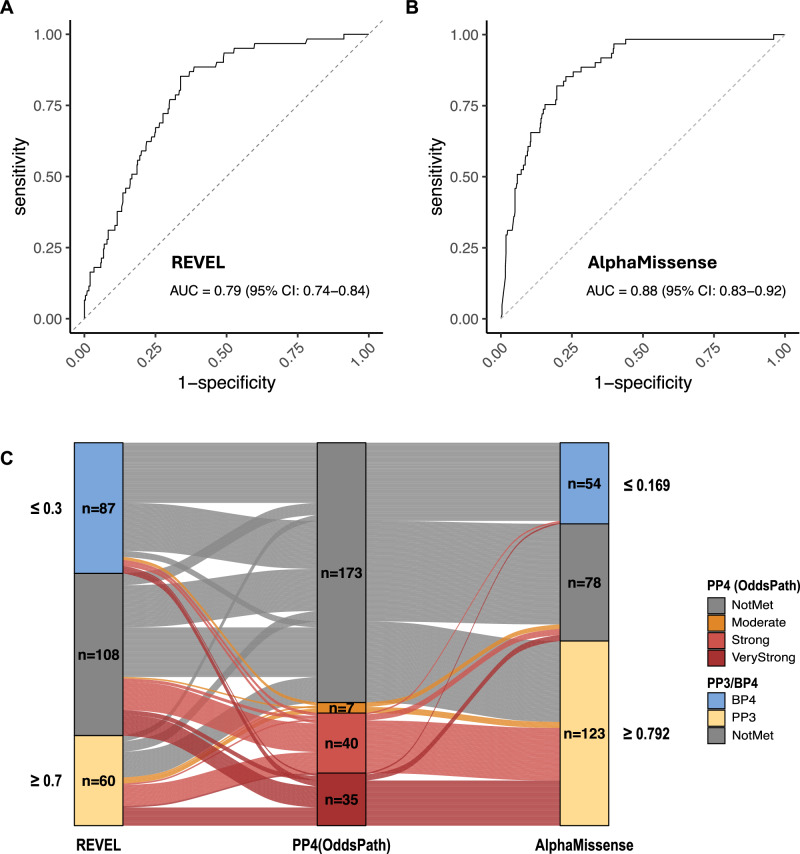
Comparison of REVEL and AlphaMissense in silico tools. The ability to classify putative pathogenic (*n* = 61) and non-pathogenic (*n* = 503) variants, based on the presence of any concurrent single recurrent somatic variant, was evaluated and compared. **A** Receiver Operating Characteristic (ROC) curve based on REVEL scores. **B** ROC curve based on AlphaMissense scores. **C** Sankey diagram illustrating the classification of variants as pathogenic supporting, benign supporting, or not met, based on REVEL (≥0.7 and ≤0.3) and AlphaMissense scores (≥0.792 and ≤0.169), across 255 evaluable missense variants with varying levels of odds of pathogenicity (OddsPath, based on the presence of somatic *DDX41* variant). Six missense variants are excluded due to missing HGVSc information (*n* = 5) or delins variant type (*n* = 1). Note that this is not the final *PP3* or *BP4* classification, which also considers the potential splicing impact (e.g., by SpliceAI score).

Using the REVEL score, the receiver operating characteristic (ROC) curve demonstrated an area under the curve (AUC) of 0.79 (95% CI: 0.74–0.84), and the optimal REVEL score threshold (Youden’s index) was 0.33 (Fig. 5A). The performance of three REVEL thresholds (≥0.33, 0.64 [37] and 0.70 [3]) was compared in Table S5.

We then evaluated AlphaMissense’s ability to identify putative pathogenic variants. Alpha scores outperformed REVEL with an AUC of 0.88 (95% CI: 0.83–0.92; *p* < 0.001 by Delong test) (Fig. 5B). The performance of three AlphaMissense thresholds (pre-determined class [36], ≥0.792 [38] and 0.91 [Youden’s index]) was compared (Table S5). We chose the AlphaMissense scores ≥0.792 and ≤0.169 for *PP3* and *BP4*, respectively, as recommended by ClinGen, for ongoing analysis. REVEL was better at identifying non-pathogenic variants, though there was significant overlap. Conversely, putative pathogenic variants clustered around high alpha scores (Fig. S9). Applied to *DDX41*-ASC (255 evaluable variants), AlphaMissense better identified variants with higher OddsPath (based on multinomial somatic hits), but had more false positives (Fig. 5C). Of 82 variants with OddsPath ≥4.33, 69 (84%) and 32 (39%) met *PP3* by AlphaMissense and REVEL. Of 173 variants with OddsPath <2.08, 52 (30%) and 76 (44%) met *BP4* by AlphaMissense and REVEL, respectively, while 54 (31%) and 28 (16%) met *PP3*.

### Updated DDX41 variant classification

**Fig. 6:**
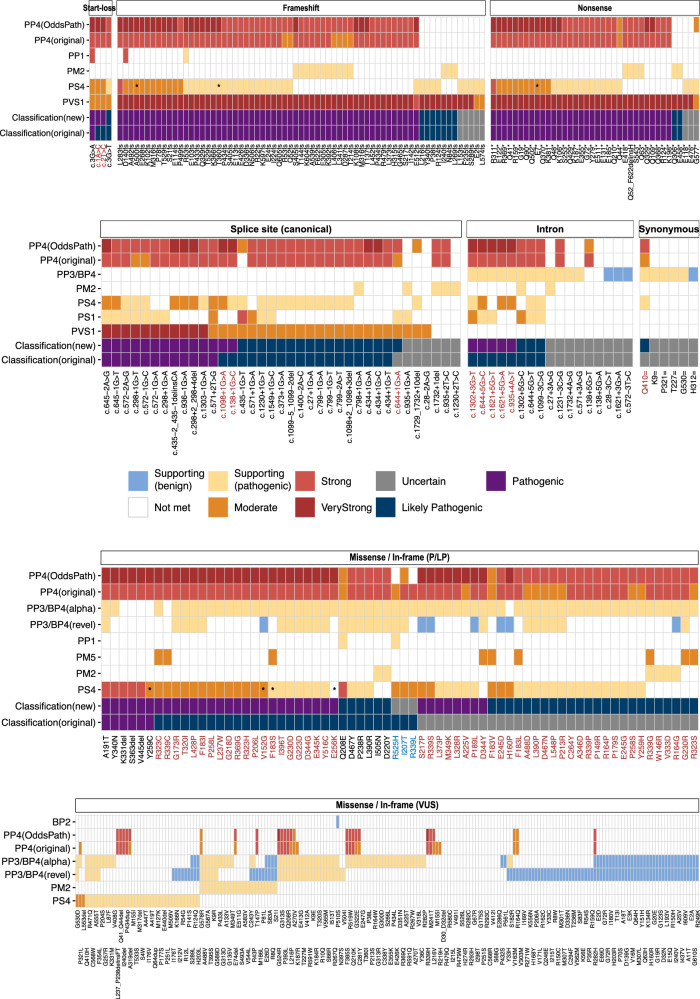
Summary of *DDX41* variant curation based on modified ACMG/AMP criteria. Each of the 438 variants is shown on the x-axis (using abbreviated nomenclature), including start-loss (*n* = 4), frameshift (*n* = 67), nonsense (*n* = 38), canonical splice site (*n* = 37), intronic (*n* = 17), synonymous (*n* = 6), missense (*n* = 66) or in-frame (*n* = 3) pathogenic/likely pathogenic (P/LP), and missense (*n* = 190) or in-frame (*n* = 10) variants of uncertain significance (VUS). Twelve variants were excluded: structural (*n* = 6), untranslated region (*n* = 1), and missing variant information (*n* = 5 missense variants). The applicable ACMG/AMP criteria are shown in each row, including the comparison between two *PP4* approaches (modified from Maierhofer et al. [3]. [PP4(original)] versus odds of pathogenicity from multinomial probability [PP4(OddsPath)]) and *PP3/BP4* approaches (REVEL [PP3/BP4(revel)] versus AlphaMissense [PP3/BP4(alpha)]). The asterisks (*) on *PS4* indicate the revised strength of evidence based on matching for East Asian genetic ancestry. Comparisons are made between the original and updated pathogenicity classifications, with red and blue text indicating upgraded and downgraded variants, respectively. Note that five variants have two different HGVSc descriptions (listed from left to right in the order of appearance) and are shown twice: R53fs (c.155dup, c.156_157insA); M316fs (c.947_948del, c.946_947del); T529fs (c.1585dup, c.1586_1587del); G72R (c.214G>A, c.214G>C); and M155I (c.465G>C, c.465G>A).

Finally, we integrated the above analysis to classify 438 evaluable germline *DDX41* variants (Fig. 6). A total of 65 variants were upgraded: 34 from VUS (26 to LP and 8 to P), and 30 from LP to P. These included 32 missense variants initially classified as VUS, based on a combination of high OddsPath from observed somatic hits (*PP4*), supporting-to-moderate level of case enrichment (*PS4*) in MDS/AML, and predicted deleterious effects by AlphaMissense (*PP3*). Thirty-two variants with high *PP4*_OddsPath (8 moderate, 22 strong, and 2 very strong) remained classified as VUS, particularly affecting missense (*n* = 21) and in-frame (*n* = 5) variants, due to the lack of other applicable criteria.

We created an automated application that interfaces with the *DDX41*-ASC to support classification of pathogenicity according to ACMG/AMP criteria (https://blombery-lab.shinyapps.io/ddx41). In addition to calculating the odds ratio for case enrichment and OddsPath based on observed somatic hits, the application also features a customizable user interface, allowing users to specify disease contexts, second somatic hotspots, in silico tools for *PP3/BP4* (REVEL or AlphaMissense), and thresholds for various curation criteria such as *PS4*, REVEL [35], SpliceAI [39], and population allele frequency. Each queried variant provides detailed information, including relevant literature and related somatic hits. Users can also manually override the criteria for pathogenicity classification. The application supports bulk curation of variants, enabling multiple variants to be curated simultaneously with the standardized application of pre-specified rules.

## Discussion

Evidence-based and reproducible classification of *DDX41* variants by clinical molecular pathology laboratories/services and researchers is critical for optimal patient management. To this end, we have comprehensively aggregated existing published data into a large synthetic cohort comprising 54518 total patients, 2628 total germline and somatic *DDX41* cases, and 450 unique germline *DDX41* variants. The *DDX41*-ASC has enabled analyses that have provided insights informing the evidence of pathogenicity as per existing variant curation frameworks (ACMG/AMP). An online curation tool was developed to query germline *DDX41* variants, retrieve literature cases and somatic hit data, and apply standardized, yet customizable, curation rules for consistent interpretation across laboratories with a single click.

A hallmark of cancer predisposition genes is the significant enrichment in patients with a given phenotype compared to matched controls. Given the relatively high frequency of *DDX41* variants in the population due to their minimal impact on reproductive fitness, very large case-control studies are required to study disease associations effectively. Although several large MDS/AML cohorts have been documented in the literature, gathering sufficient evidence to meet this criterion (*PS4*) requires labor-intensive manual review of publications. Furthermore, because founder variants are more common in certain ancestry groups, unadjusted case-control comparisons may overestimate enrichment. The creation of a *DDX41*-ASC enables the reproducible assessment of *DDX41* variants with improved statistical accuracy.

One key characteristic of myeloid malignancy in the context of *DDX41*-related hematologic malignancy predisposition syndrome is the presence of a second somatic variant in *DDX41*. Several groups have relied on the presence of either recurrent (variously defined) or any somatic hit to inform variant pathogenicity [1, 8, 40, 41]. Our previous work demonstrated a highly non-random co-occurrence between deleterious germline and somatic *DDX41* variants (posterior probability 99.8%), suggesting that such findings could help strengthen the *PP4* criterion to a very strong level [3]. Current work refines the approach by incorporating the frequency with which a germline variant co-occurs with different (multinomial) somatic patterns, updating posterior probability and OddsPath dynamically. This method tackles the inevitable issue of somatic *DDX41* variants coinciding with a given germline *DDX41* variant, preventing false attribution of pathogenicity.

The use of computational (in silico) prediction tools, although mainly providing minor evidence of pathogenicity, can still significantly impact the final variant classification. Our analysis revealed that the commonly used REVEL score thresholds lacked sufficient sensitivity for identifying putative pathogenic missense variants in *DDX41*, resulting in under-classification in many instances. In contrast, AlphaMissense—a newer deep learning-based model—demonstrated superior diagnostic performance. Implementing AlphaMissense caused a significant shift in both *PP3* and *BP4* calls, with more variants reaching the *PP3* threshold. This increased sensitivity did not compromise specificity when combined with other criteria, as no putative benign variants (lacking recurrent somatic hits) were incorrectly classified as P/LP.

Our work has several important limitations to acknowledge. This work relied on cases reported in the literature instead of re-analysis of raw sequencing data. Despite the large size of the aggregated cohort, data on non-MDS/AML cases, including lymphoid neoplasms, are limited and preclude any meaningful conclusions regarding the association between *DDX41* germline predisposition and these entities. The absence of detailed ancestry information in many source publications hampers more precise quasi-case-control analysis. Most available data come from individuals of NFE and EAS ancestries, which limits the generalizability of our findings to other populations and highlights the need for more data from diverse ancestry groups. The variant and clinical data were manually extracted from various publications that vary in nomenclature, sequencing platforms, bioinformatic pipelines, and reporting standards. Therefore, intronic and structural variant types are almost certainly underrepresented. The frequency and number of concurrent somatic mutations would also vary with detection sensitivity. Most publications assumed the germline versus somatic origin of the *DDX41* variants rather than basing this on direct evidence from paired testing with non-hematological tissue; misclassification could significantly affect the OddsPath, especially in rare cases. Since the model relies on data from known deleterious variants, it is not suitable for variants with lower penetrance or different somatic association patterns; further work is needed to refine the approach [42].

In summary, by creating a *DDX41*-ASC and related analyses, we have made multiple refinements in variant classification. These include an ancestry-matched quasi-case-control study for a more precise assessment of case enrichment, enhanced sophistication in incorporating *DDX41* somatic variants into classification, and the identification of AlphaMissense as a potentially preferred computational tool for pathogenicity assessment over REVEL. Ongoing collaboration and data sharing will be essential to refine these recommendations further and help incorporate them into standard diagnostic practice, facilitated by a publicly available online tool, and to include non-European, non-East Asian groups, such as African and South Asian datasets. Our analysis of germline-somatic pairs and somatic VAFs may guide future functional studies in this germline predisposition entity.

We look forward to the incorporation of our work into ClinGen-approved *DDX41* variant curation rules, which can be implemented broadly in clinical laboratories worldwide.

## Supplementary information

Supplementary Methods

Figure S1

Figure S2

Figure S3

Figure S4

Figure S5

Figure S6

Figure S7

Figure S8

Figure S9

Table S1A

Table S1B

Table S2

Table S3

Table S4

Table S5

## Data Availability

Code available at https://github.com/blomberylab/ddx41.
