# Supplementary material for: Refinement of the classification of DDX41 variants through analysis of aggregated clinical datasets
Source: Leukemia. 2026 Feb 17;40(3):649–60. doi: 10.1038/s41375-026-02886-6 (PMC12960222; doi:10.1038/s41375-026-02886-6)
Supplement: Supplementary file 2 — Figure S1 [file 41375_2026_2886_MOESM2_ESM.pdf]

Figure S1

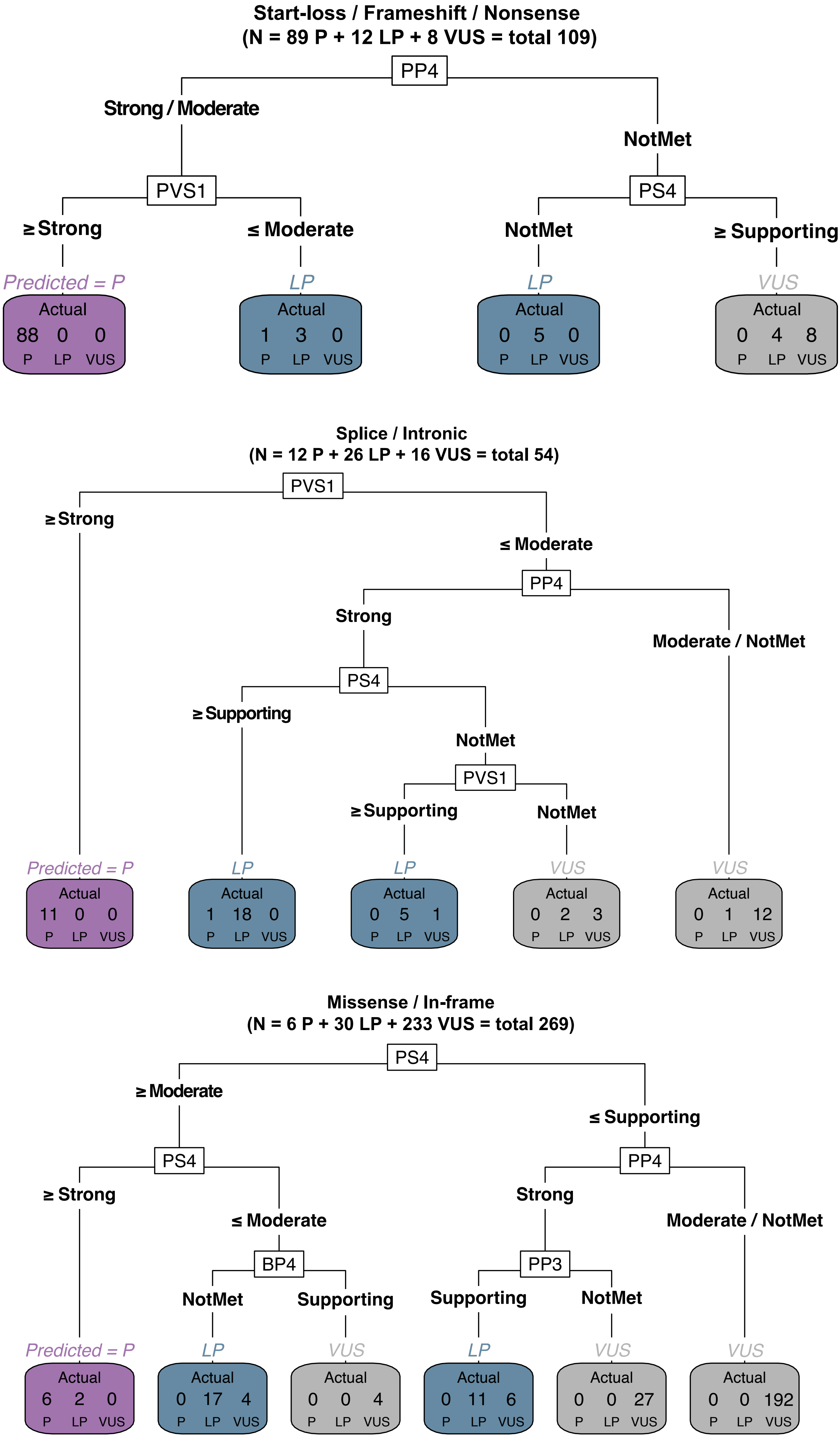
Start-loss / Frameshift / Nonsense
(N = 89 P + 12 LP + 8 VUS = total 109)
PP4
Strong / Moderate
NotMet
PVS1
≥ Strong
≤ Moderate
PS4
NotMet
≥ Supporting
Predicted = P
Actual
88 0 0
P LP VUS
LP
Actual
1 3 0
P LP VUS
LP
Actual
0 5 0
P LP VUS
VUS
Actual
0 4 8
P LP VUS
Splice / Intronic
(N = 12 P + 26 LP + 16 VUS = total 54)
PVS1
≥ Strong
≤ Moderate
PP4
Strong
Moderate / NotMet
PS4
≥ Supporting
NotMet
PVS1
≥ Supporting
NotMet
Predicted = P
Actual
11 0 0
P LP VUS
LP
Actual
1 18 0
P LP VUS
LP
Actual
0 5 1
P LP VUS
VUS
Actual
0 2 3
P LP VUS
VUS
Actual
0 1 12
P LP VUS
Missense / In-frame
(N = 6 P + 30 LP + 233 VUS = total 269)
PS4
≥ Moderate
≤ Supporting
PS4
≥ Strong
≤ Moderate
BP4
NotMet
Supporting
PP4
Strong
Moderate / NotMet
PP3
Supporting
NotMet
Predicted = P
Actual
6 2 0
P LP VUS
LP
Actual
0 17 4
P LP VUS
VUS
Actual
0 0 4
P LP VUS
LP
Actual
0 11 6
P LP VUS
VUS
Actual
0 0 27
P LP VUS
VUS
Actual
0 0 192
P LP VUS

Figure S1. Recursive partitioning decision tree of DDX41 germline variant classification. Variants are grouped by type: (A) start-loss, frameshift, and nonsense variants (n=109); (B) canonical splice site and intronic variants (n=54); and (C) missense and in-frame variants (n=269). Five missense variants lacking HGVSc information with multiple possible backtranslations were excluded from the analysis. The decision tree is generated based on all ACMG/AMP criteria using the rpart package. The hierarchical splits represent sequential criteria, with higher splits indicating more influential factors in the initial decision-making process. Variants are classified into Pathogenic (P, purple), Likely Pathogenic (LP, blue), or Variant of Uncertain Significance (VUS, gray) based on the algorithm's predictive classification. Each terminal node contains three numbers representing the actual counts of variants with P, LP, and VUS classifications, as described in the Supplemental Methods. Recursive partitioning is a criterion-based analysis for variant type rather than individual variants; therefore, a discrepancy between the exact counts and the assigned pathogenicity class within a terminal node does not indicate a final misclassification but rather reflects the current decision-making state up to that partition.
