# Supplementary material for: Refinement of the classification of DDX41 variants through analysis of aggregated clinical datasets
Source: Leukemia. 2026 Feb 17;40(3):649–60. doi: 10.1038/s41375-026-02886-6 (PMC12960222; doi:10.1038/s41375-026-02886-6)
Supplement: Supplementary file 4 — Figure S3 [file 41375_2026_2886_MOESM4_ESM.pdf]

Figure S3

A

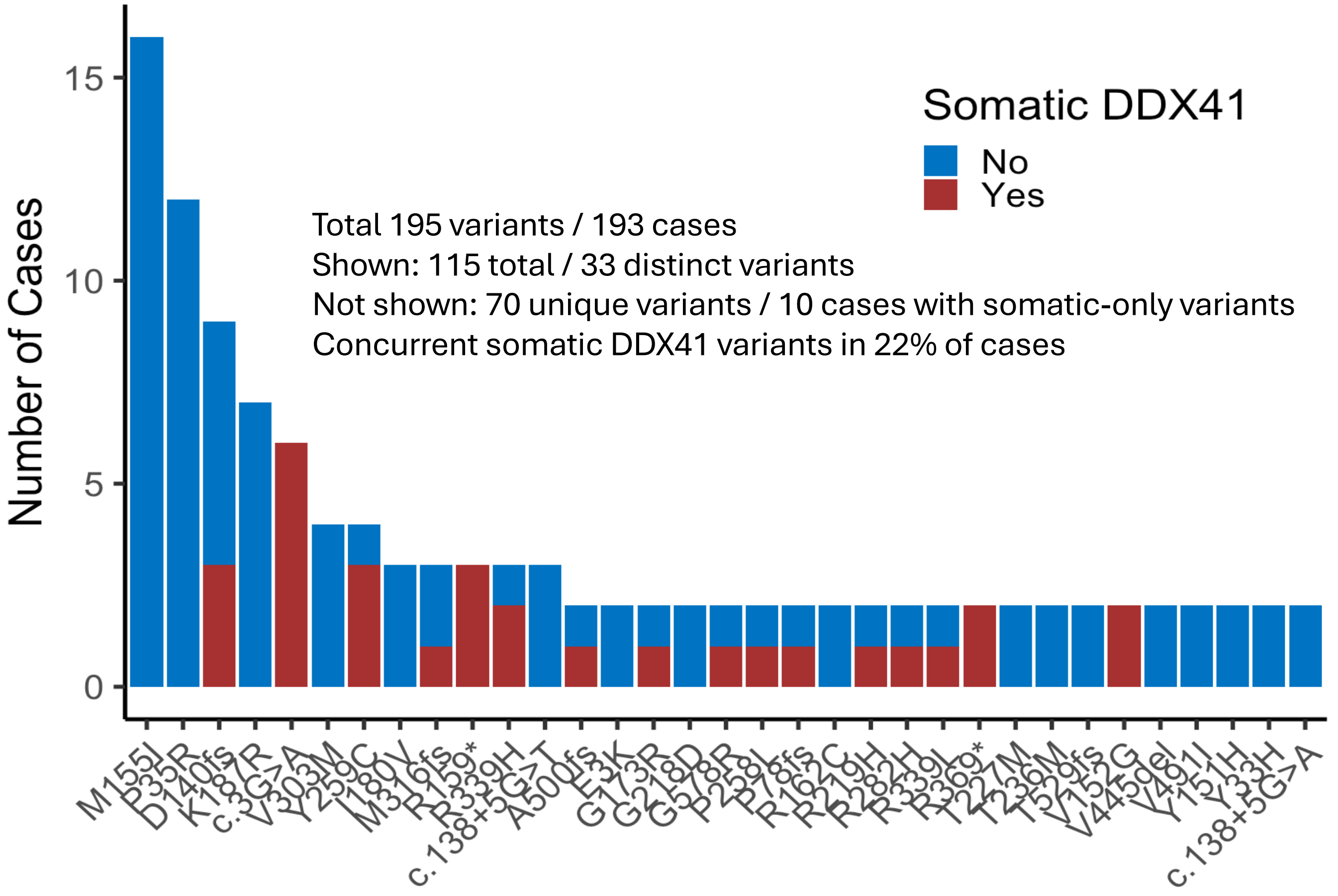

B

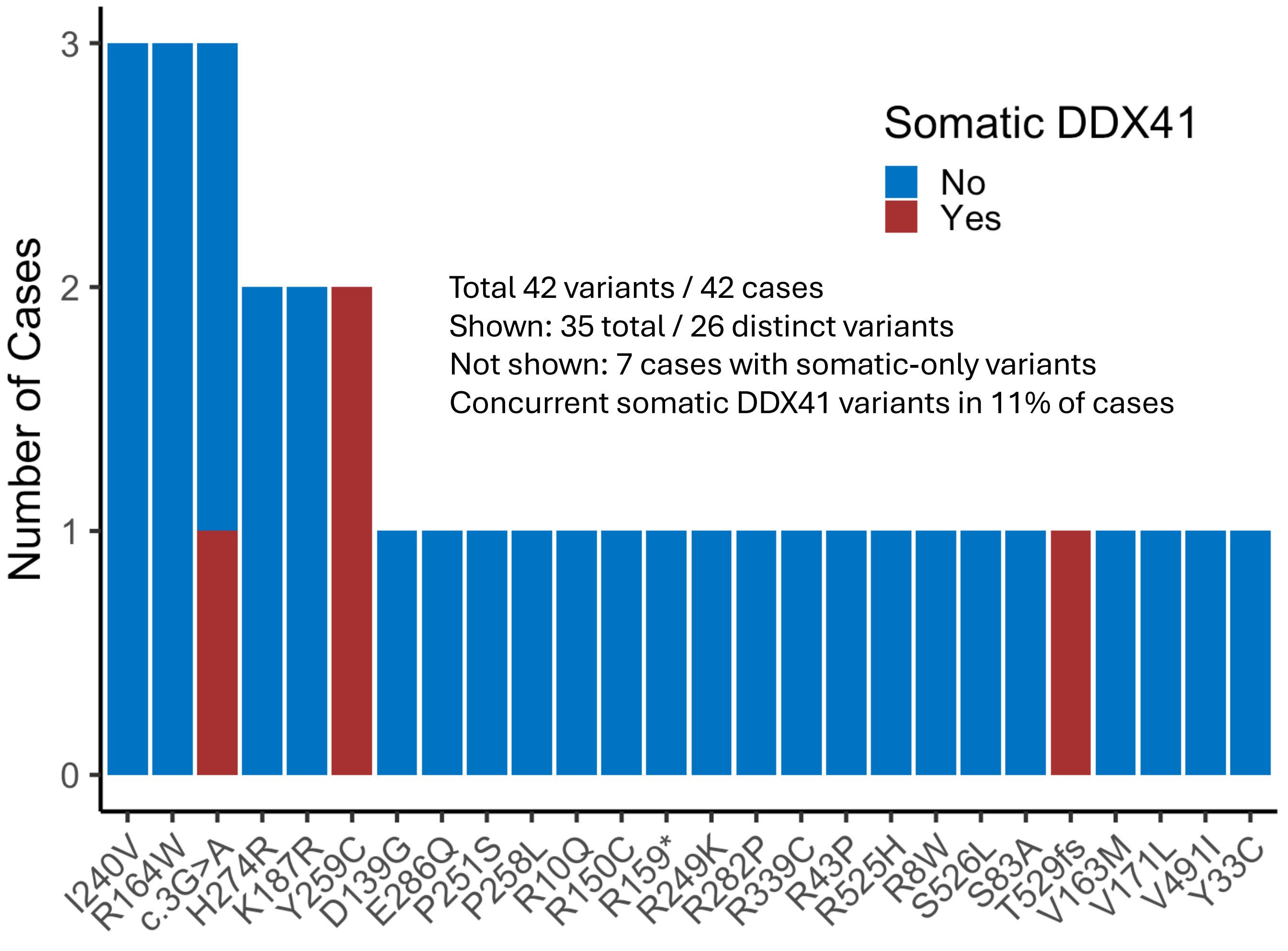

Figure S3. Spectrum of *DDX41* variants outside the context of myelodysplastic syndrome and acute myeloid leukemia (MDS/AML). (A) Non-MDS/AML myeloid neoplasms and cytopenias, and **(B)** lymphoid neoplasms. The presence of a concurrent somatic *DDX41* variant is indicated with red (yes) or blue (no).
