# Supplementary material for: Refinement of the classification of DDX41 variants through analysis of aggregated clinical datasets
Source: Leukemia. 2026 Feb 17;40(3):649–60. doi: 10.1038/s41375-026-02886-6 (PMC12960222; doi:10.1038/s41375-026-02886-6)
Supplement: Supplementary file 5 — Figure S4 [file 41375_2026_2886_MOESM5_ESM.pdf]

**Figure S4**

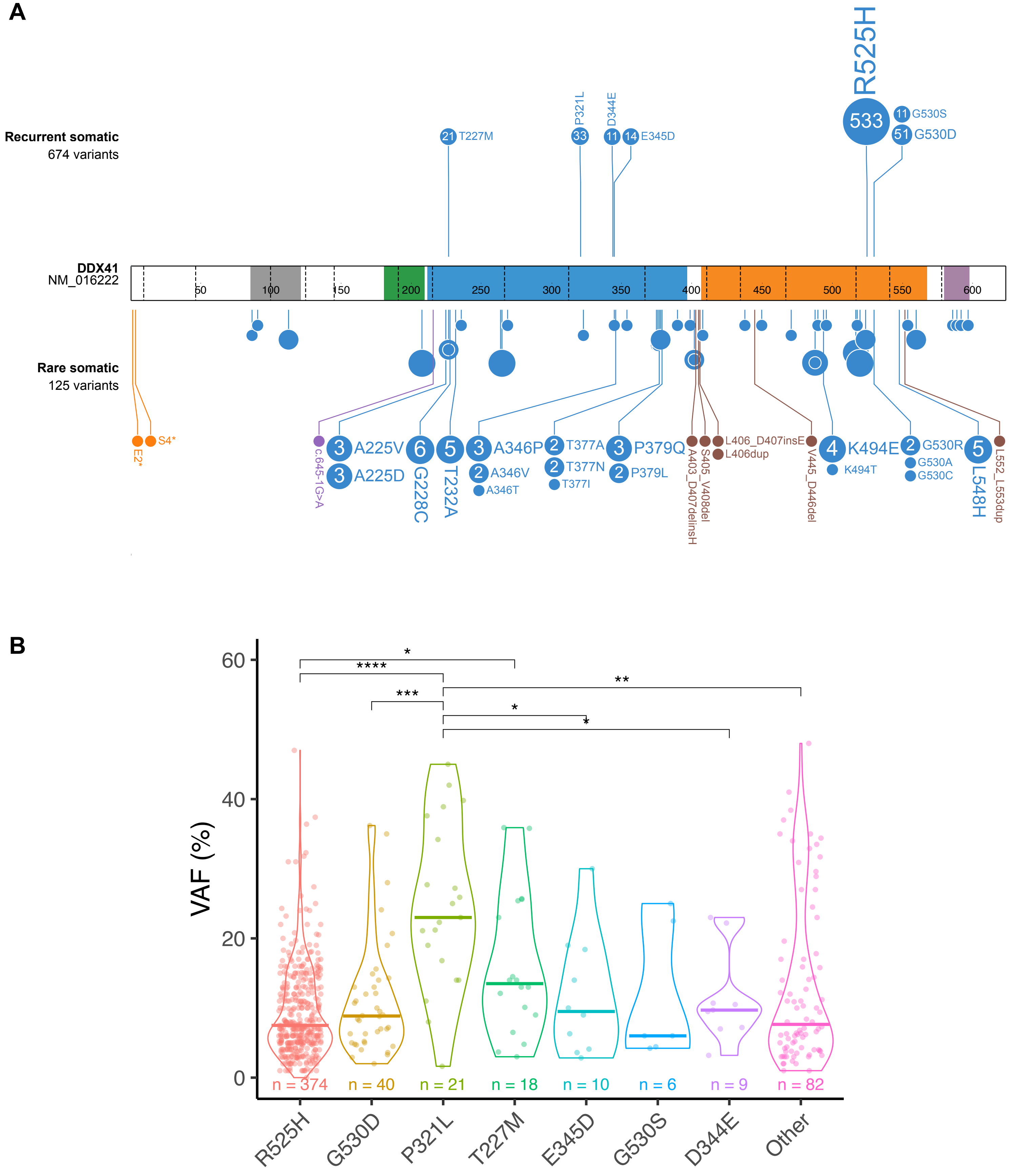


**Figure S4. Summary of single *DDX41* somatic variants** observed alongside a germline DDX41 variant in 799 myelodysplastic syndrome/acute myeloid leukemia (MDS/AML) cases. One case was excluded due to missing variant information. (A) The top seven recurrent somatic variants (top) constituted 674 (84%) of the cases. Rare somatic variants (bottom) included 75 different variants found in 125 (16%) cases; all were missense or in-frame variants except for three: E2* (variant allele fraction [VAF] unknown), S4* (VAF unknown), and c.645-1G>A (VAF 1%), which were all identified alongside the germline R369G variant (in separate cases). (B) Violin and dot plot of VAFs among (primarily assumed) somatic DDX41 variants, showing significant differences between groups (p=2.2e-6 by Kruskal-Wallis test). Pairwise Wilcoxon tests were adjusted using the Benjamini-Hochberg method. P-value annotations: <0.05 (*), <0.01 (**), <0.001 (***), <0.0001 (****).
