# Supplementary material for: Refinement of the classification of DDX41 variants through analysis of aggregated clinical datasets
Source: Leukemia. 2026 Feb 17;40(3):649–60. doi: 10.1038/s41375-026-02886-6 (PMC12960222; doi:10.1038/s41375-026-02886-6)
Supplement: Supplementary file 6 — Figure S5 [file 41375_2026_2886_MOESM6_ESM.pdf]

Figure S5

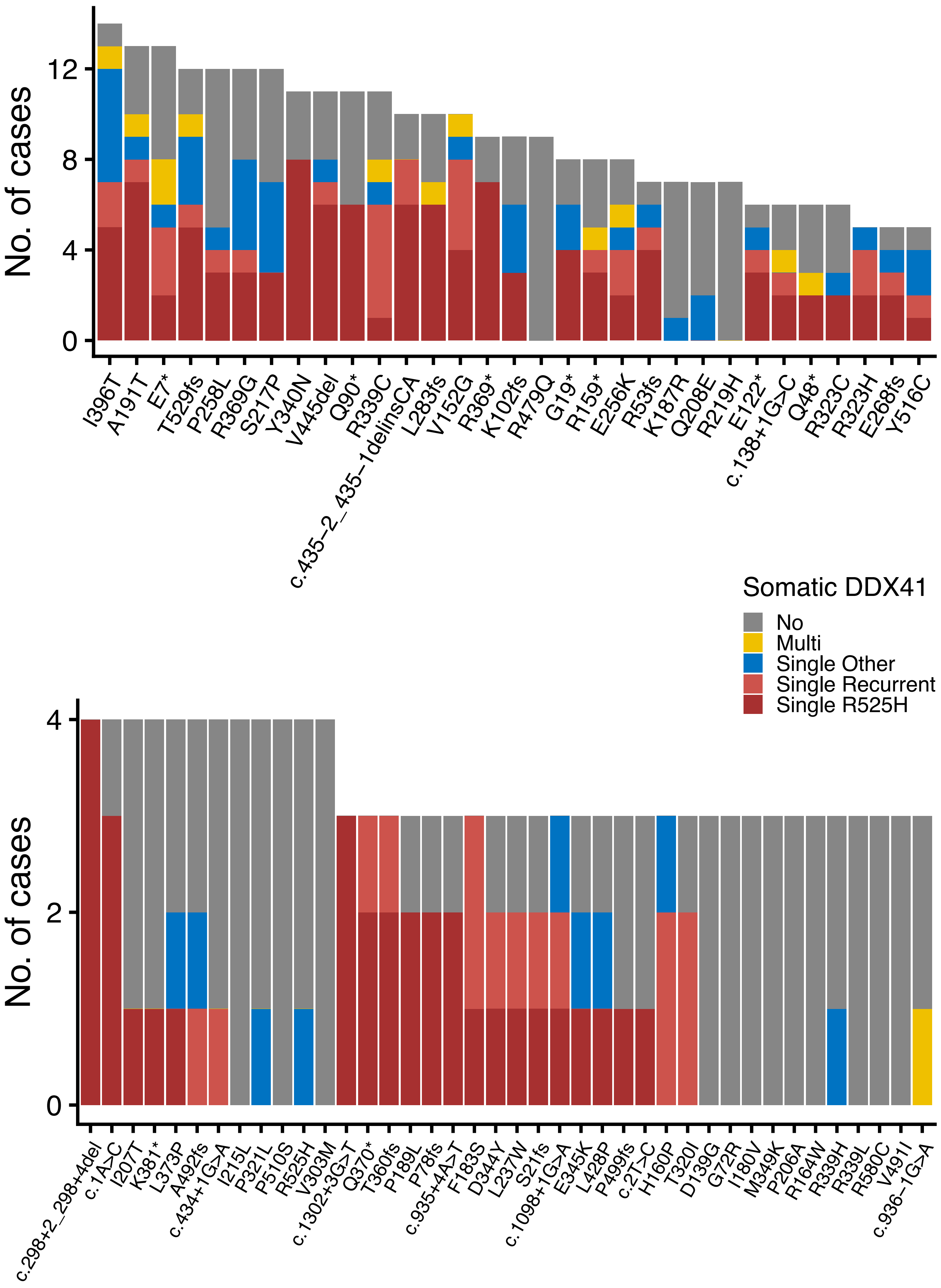

Figure S5. Association between less common germline *DDX41* variants and somatic variant types. The top panel shows germline variants with 5 to 14 occurrences, while the bottom panel displays those with 3 to 4 occurrences.
