# Supplementary material for: Refinement of the classification of DDX41 variants through analysis of aggregated clinical datasets
Source: Leukemia. 2026 Feb 17;40(3):649–60. doi: 10.1038/s41375-026-02886-6 (PMC12960222; doi:10.1038/s41375-026-02886-6)
Supplement: Supplementary file 7 — Figure S6 [file 41375_2026_2886_MOESM7_ESM.pdf]

Figure S6

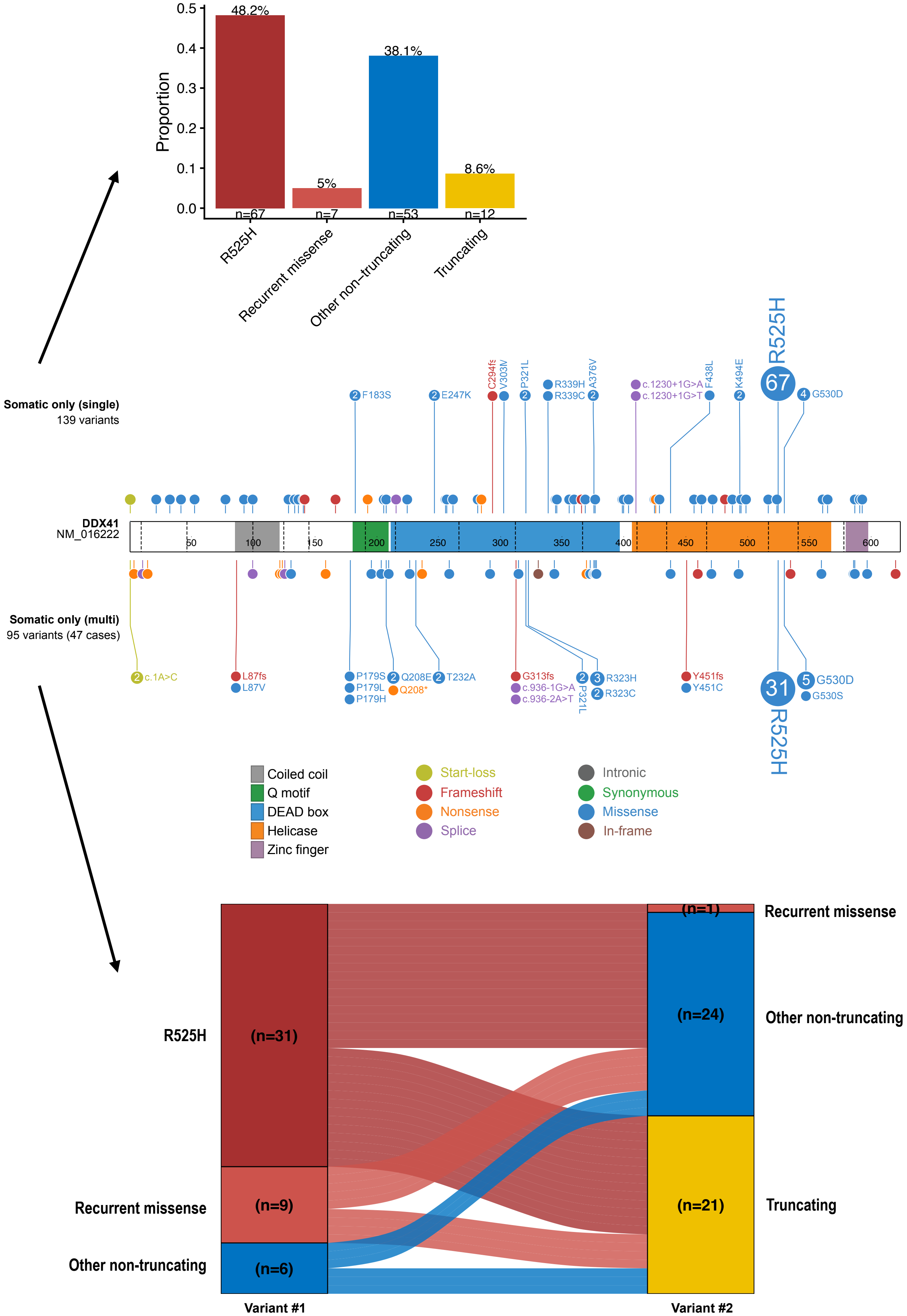

Figure S6. Characteristics of somatic-only *DDX41* variants. The middle panel presents a summary of 139 single variants and 95 multiple somatic variants identified in 47 cases (one case had three variants). The top panel summarizes the different types of single somatic variants, while the bottom panel illustrates the relationship between pairs of variants in the 46 cases with double (assumed) somatic-only DDX41 variants.
