# Supplementary material for: Refinement of the classification of DDX41 variants through analysis of aggregated clinical datasets
Source: Leukemia. 2026 Feb 17;40(3):649–60. doi: 10.1038/s41375-026-02886-6 (PMC12960222; doi:10.1038/s41375-026-02886-6)
Supplement: Supplementary file 8 — Figure S7 [file 41375_2026_2886_MOESM8_ESM.pdf]

Figure S7

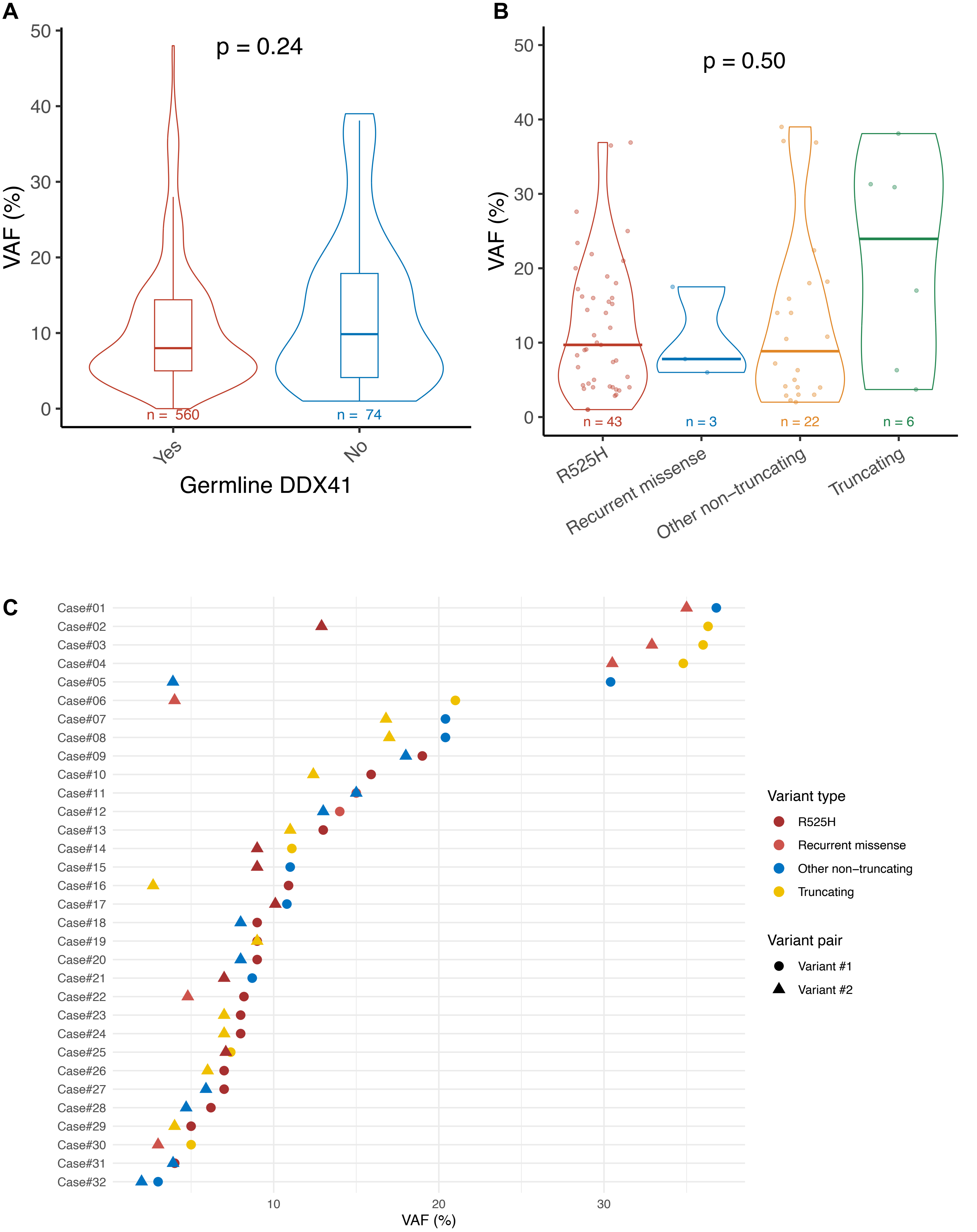

Figure S7. Variant allele fractions (VAF %) of somatic-only *DDX41* variants. (A) Comparison of VAFs between single somatic *DDX41* variants in cases with and without a germline variant. **(B)** VAFs of single somatic-only *DDX41* variants based on variant types. **(C)** VAFs of double (assumed) somatic-only *DDX41* variants. Variants #1 (circle) and #2 (triangle) represent the higher and lower VAF variants, respectively, and the colors indicate the variant types.
