# Supplementary material for: Refinement of the classification of DDX41 variants through analysis of aggregated clinical datasets
Source: Leukemia. 2026 Feb 17;40(3):649–60. doi: 10.1038/s41375-026-02886-6 (PMC12960222; doi:10.1038/s41375-026-02886-6)
Supplement: Supplementary file 9 — Figure S8 [file 41375_2026_2886_MOESM9_ESM.pdf]

Figure S8

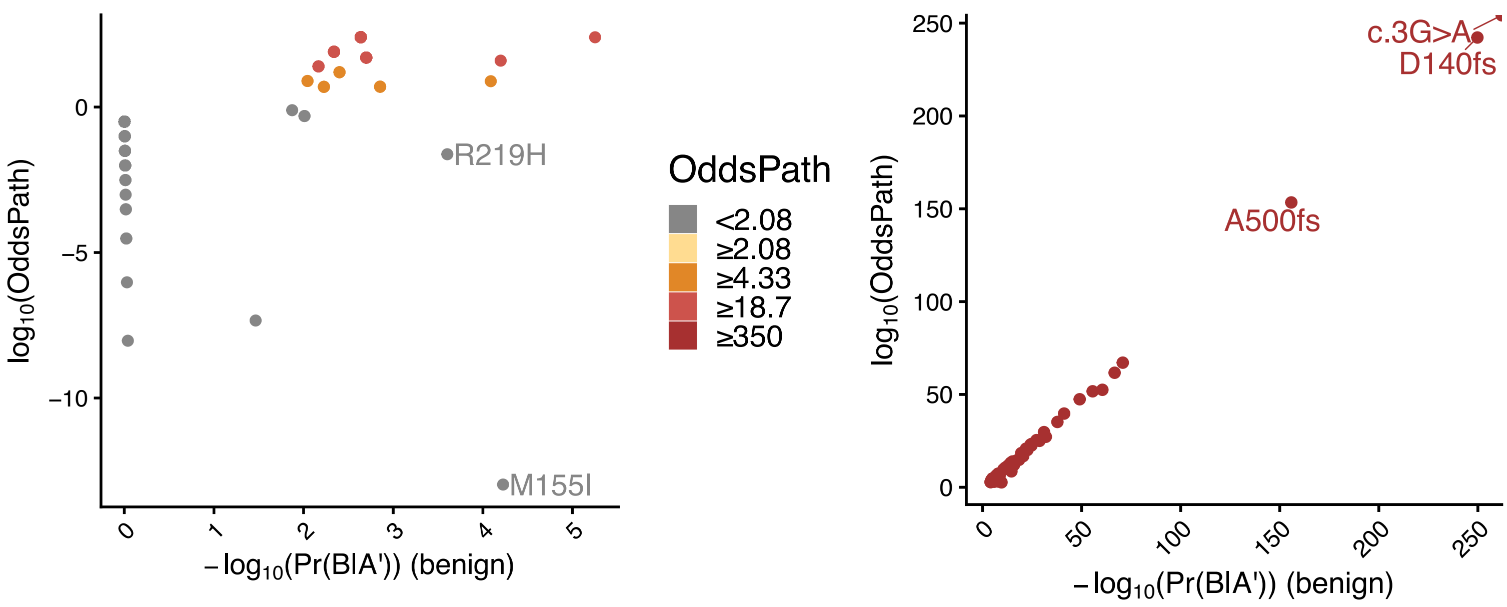

Figure S8. The relationship between odds of pathogenicity (OddsPath) and Pr(B|A') for each germline *DDX41* variant. Pr(B|A') is the probability of observed somatic *DDX41* patterns by chance without a germline deleterious *DDX41* variant. OddsPath, derived from the posterior probability, is applied to the PP4 criterion. The x-axis is in a negative log10 scale: larger values indicate smaller Pr(B|A'). Variants with very strong OddsPath (≥350) are illustrated on the right panel, while those with OddsPath <350 are on the left panel.
