# Supplementary material for: Refinement of the classification of DDX41 variants through analysis of aggregated clinical datasets
Source: Leukemia. 2026 Feb 17;40(3):649–60. doi: 10.1038/s41375-026-02886-6 (PMC12960222; doi:10.1038/s41375-026-02886-6)
Supplement: Supplementary file 10 — Figure S9 [file 41375_2026_2886_MOESM10_ESM.pdf]

Figure S9

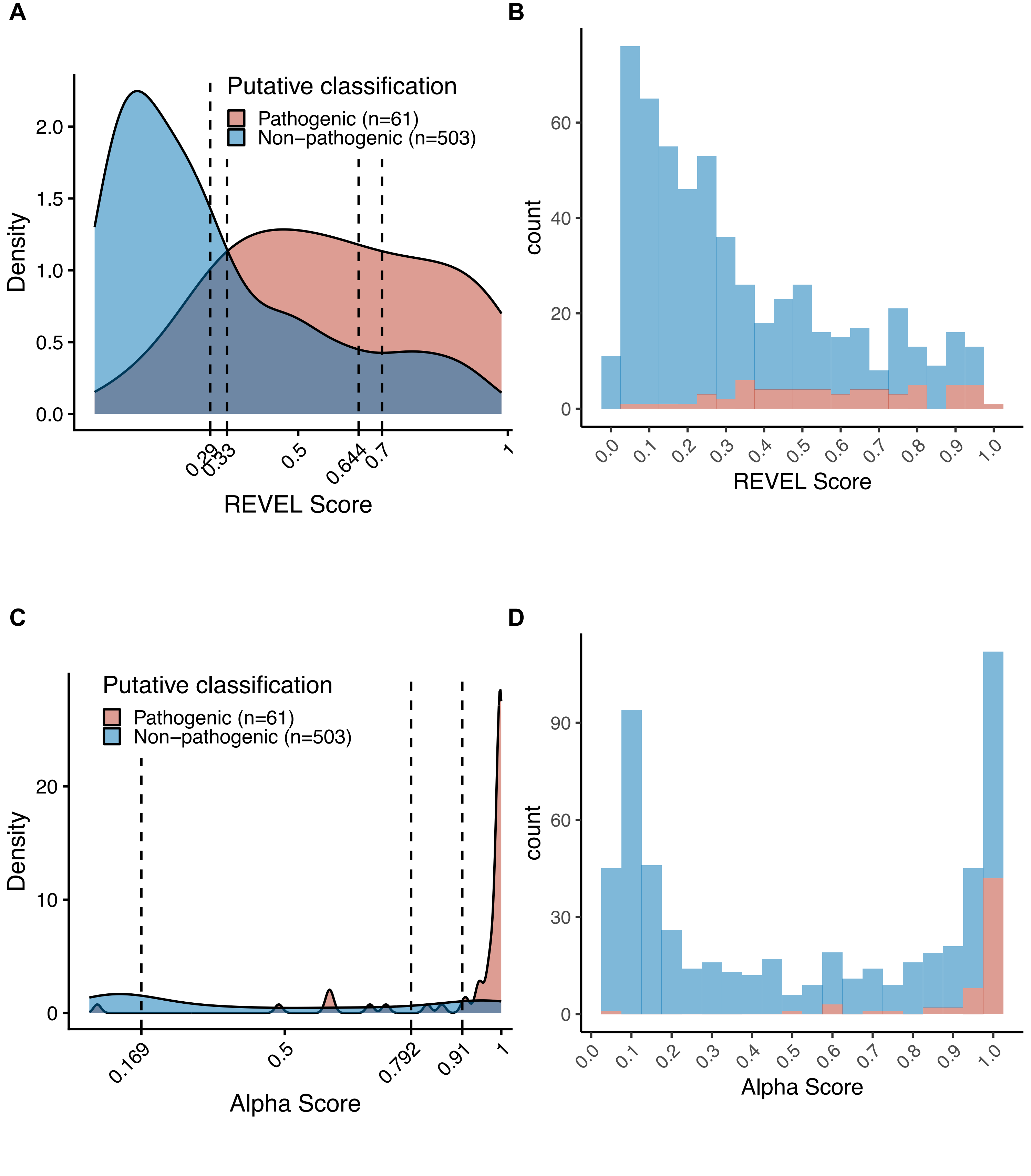

Figure S9. Comparison of REVEL and AlphaMissense in silico tools. The ability to classify putative pathogenic (n=61, red) and non-pathogenic (n=503, blue) variants, based on the presence of any concurrent single recurrent somatic variant, was evaluated and compared. **(A)** Density plot and **(B)** histogram of REVEL scores in the variant classification. **(C)** Density plot and **(D)** histogram of AlphaMissense in the variant classification. Dashed vertical lines indicate various REVEL or AlphaMissense score thresholds.
